# Effect of molecular crowding on the biological identity of liposomes: an overlooked factor at the bio-nano interface

**DOI:** 10.1039/c9na00195f

**Published:** 2019-05-31

**Authors:** Luca Digiacomo, Francesca Giulimondi, Morteza Mahmoudi, Giulio Caracciolo

**Affiliations:** Department of Molecular Medicine, Sapienza University of Rome Viale Regina Elena 291 00161 Rome Italy giulio.caracciolo@uniroma1.it; Department of Anesthesiology, Brigham and Women's Hospital, Harvard Medical School Boston MA 02115 USA MMAHMOUDI@bwh.harvard.edu

## Abstract

Once embedded in a physiological environment, the surface of nanoparticles (NPs) gets covered with a biomolecular corona (BC) that alters their synthetic characteristics and subsequently gives them a peculiar biological identity. Despite recent studies having clarified the role of NP composition, surface chemistry and biological source (*e.g.*, human/animal serum or plasma) in the formation of the BC, little is known about the possible impact of molecular crowding. To fill this gap, we used a cationic liposomal formulation as a model system and studied its biological identity upon incubation with human plasma, at a fixed liposome-to-plasma volume ratio and different concentrations. We carried out dynamic light scattering measurements to quantify the size and zeta potential of the investigated systems and gel electrophoresis to evaluate the composition of the corresponding coronas. Our findings suggest that NP stability may be compromised by molecular crowding, but the corona composition is stable over a wide range of concentrations, which extend over more than two orders of magnitude. As the biological identity of NPs eventually determines their final fate *in vivo*, we predict that this study could contribute to the development of a safe and effective nanosystem for the targeted delivery of therapeutic agents.

## Abstract 2

Once embedded in a physiological environment, the aggregation state of liposomes is deeply affected by the protein concentration.

## Graphical abstract

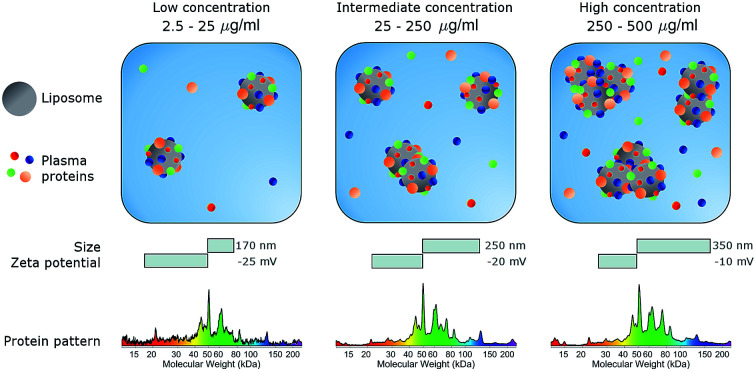

Due to their intrinsic structure, biocompatibility, and ease of preparation and functionalization, liposomes are regarded as one of the most promising classes of organic nanoparticles (NPs) for drug and gene delivery applications.^1,2^ The amphiphilic nature of their components makes liposomes an ideal platform for the encapsulation of both hydrophobic and hydrophilic molecules, including a large variety of drugs, nucleic acids, proteins and imaging agents.^3^ This structural versatility of liposomes renders the design of systems destined for specific needs practically limitless,^4^ such as vectors for gene and siRNA therapy,^5,6^ genetic vaccines,^7^ immunomodulation,^8^ and tumor targeting.^9^ To this end, liposomes are usually functionalized with appropriate ligands in order to address specifically the delivery of their cargo to the desired target cells. Grafting poly-(ethylene glycol) (PEG) onto the liposome surface prolongs their blood residency time^1^ and the synthetic modification of a terminal PEG molecule with peptides or monoclonal antibodies^1^ promotes selective accumulation in tumor regions.^9,10^ Despite the great potential of liposomes in nanomedicine, it has been elucidated that the properties of pristine NPs can be severely altered when they come into contact with biological media,^11,12^*e.g.* blood or plasma. Indeed the formation of a biomolecular layer (or corona) changes the original size, surface charge, aggregation state, and surface properties of NPs.^13,14^ In other words, the presence of a biomolecular corona (BC) turns the “synthetic identity” of NPs into a new “biological identity” that ultimately determines their biological fates including their pharmacokinetics.^12–17^ As the corona represents the biological interface that mediates the interactions between NPs and living cells, it strongly affects the NPs’ efficiency as a carrier and release platform of therapeutic agents.^18,19^ In fact, the BC plays a key role in many biological processes, *e.g.* cell internalization, endocytic pathways and intracellular trafficking.^16,20–22^ For these reasons, scientific interest in the BC has been increasing over the years and different studies have elucidated the role of NPs’ physicochemical properties, environmental conditions (*e.g.*, incubation temperature), and biomolecular sources (*e.g.*, human serum *vs.* human plasma; human plasma *vs.* mouse plasma *etc.*) in the biological identity of NPs.^12,17,23,24^ Although several ignored factors (*e.g.*, personalized protein corona,^25^ plasma concentration,^26^ incubation temperature,^27^ local temperature induced by plasmonic NPs,^28^ metabolome corona,^29^ cell vision,^30^ and cell sex^31^) have been introduced into the nano-bio interfaces community, there is still a lack of knowledge about the possible impact of molecular crowding on the formation of the BC, *i.e.* the potential variation of kinetic equilibrium among bound and unbound proteins in diluted media, with respect to the corresponding behavior under standard conditions.

Molecular crowding may be relevant for both conceptual and technical reasons. Indeed, upon extravasation from blood circulation, NPs experience different environments, which usually have lower amounts of proteins^32^ (*e.g.* the interstitial fluid). Thus, the study of the evolution of NP–BC complexes from crowded to diluted media aims to evaluate the stability and features of the systems under very specific physiological conditions. Furthermore, variation of the chemical–physical properties due to molecular crowding could affect part of the experimental activity that is focused on system characterization. Indeed, many techniques (*e.g.* dynamic light scattering, transfection assays *etc.*) require well-established protocols, sometimes involving dilution processes, which therefore could generate misleading results.

Recently, new perspectives in the use of liposome–BC for diagnostic purposes have also arisen. The hypothesis was that the BC from the blood of human subjects is “personalized”, *i.e.* it is affected by individual changes in the concentration and structure of plasma proteins as those generated under clinical conditions. There is increasing excitement over testing new technologies aimed at finding alterations of personalized BCs. For instance, tandem mass spectrometry (MS/MS) permits the detection of alterations in protein circulating levels and biomolecule concentration at very early stages of a disease, *i.e.* when changes are undetectable by routine blood tests. This proteomic approach is successful,^33,34^ but massive screening procedures could benefit from the employment of easier and cheaper benchtop techniques.

Current research is seeking a global change in the BC (*i.e.* simultaneous change in the size, zeta-potential (*Z*_p_) and protein patterns) by combining several experimental techniques such as dynamic light scattering (DLS) and one-dimensional sodium dodecyl sulphate-polyacrylamide gel electrophoresis (1D SDS-PAGE).^35^

To shed light on this overlooked but potentially relevant aspect, we studied the biological identity (*i.e.* size, zeta-potential, aggregation state and corona composition) of a model liposomal formulation at different concentrations. We chose a single-component cationic liposome made of the cationic lipid 1,2-dioleoyl-3-trimethylammonium-propane (DOTAP), as it is of great interest for gene delivery applications: it is widely used, relatively cheap and efficient in both *in vitro* and *in vivo* applications.^36^ The size and zeta potential of the bare liposome were measured and compared to those arising upon incubation with human plasma, at different concentrations that extend over more than two orders of magnitude. Afterwards, we studied the corresponding corona composition by gel electrophoresis. We found that the size, aggregation state and surface charge of liposome–BC complexes were significantly affected by molecular crowding; however the protein composition of the corona was highly stable within the explored range of sample concentrations.

DOTAP was purchased from Avanti Polar Lipids (Alabaster, AL, USA), dissolved in chloroform and the solvent was evaporated under vacuum for 2 hours. Lipid films were hydrated with ultrapure water to a final lipid concentration of 1 mg mL^−1^ and stored at 4 °C. Finally, the obtained liposome suspension was extruded 20 times through a 0.1 μm polycarbonate filter with an Avanti mini-extruder (Avanti Polar Lipids, Alabaster, AL, USA). Liposome–BC complexes were obtained by mixing 500 μL of the liposomal dispersion with 500 μL of human plasma (purchased from Sigma Aldrich, St. Louis, MI, USA) for 1 h at 37 °C. The liposomes were exposed to a strong excess of plasma proteins (1 : 1 vol/vol) because this condition is regarded as mimetic of the equilibrium conditions that are established *in vivo*.^12,37,38^

To study the effect of a crowded environment on the biological identity of liposomes, we diluted the obtained liposome–BC solution with water and obtained different samples at the following lipid concentrations: 2.5 μg mL^−1^, 5 μg mL^−1^, 25 μg mL^−1^, 50 μg mL^−1^, 250 μg mL^−1^ and 500 μg mL^−1^. Physical–chemical characterization of liposomes and liposome–BC complexes was carried out in terms of the hydrodynamic diameter and zeta potential of the investigated systems. All size and zeta potential measurements were made on a Zetasizer Nano ZS90 (Malvern, U.K.) equipped with a 5 mW HeNe laser (wavelength = 633 nm) and a digital logarithmic correlator. Fig. 1A and B demonstrate the measured size and zeta potential distributions of bare liposomes (reference curves) and liposome–BC complexes at a fixed liposome-to-plasma ratio (1 : 1 vol/vol) and different lipid concentrations. The size distribution of bare liposomes was unimodal, centred at 131 ± 7 nm and quite narrow (polydispersity index: PdI = 0.145 ± 0.047).

**Fig. 1:**
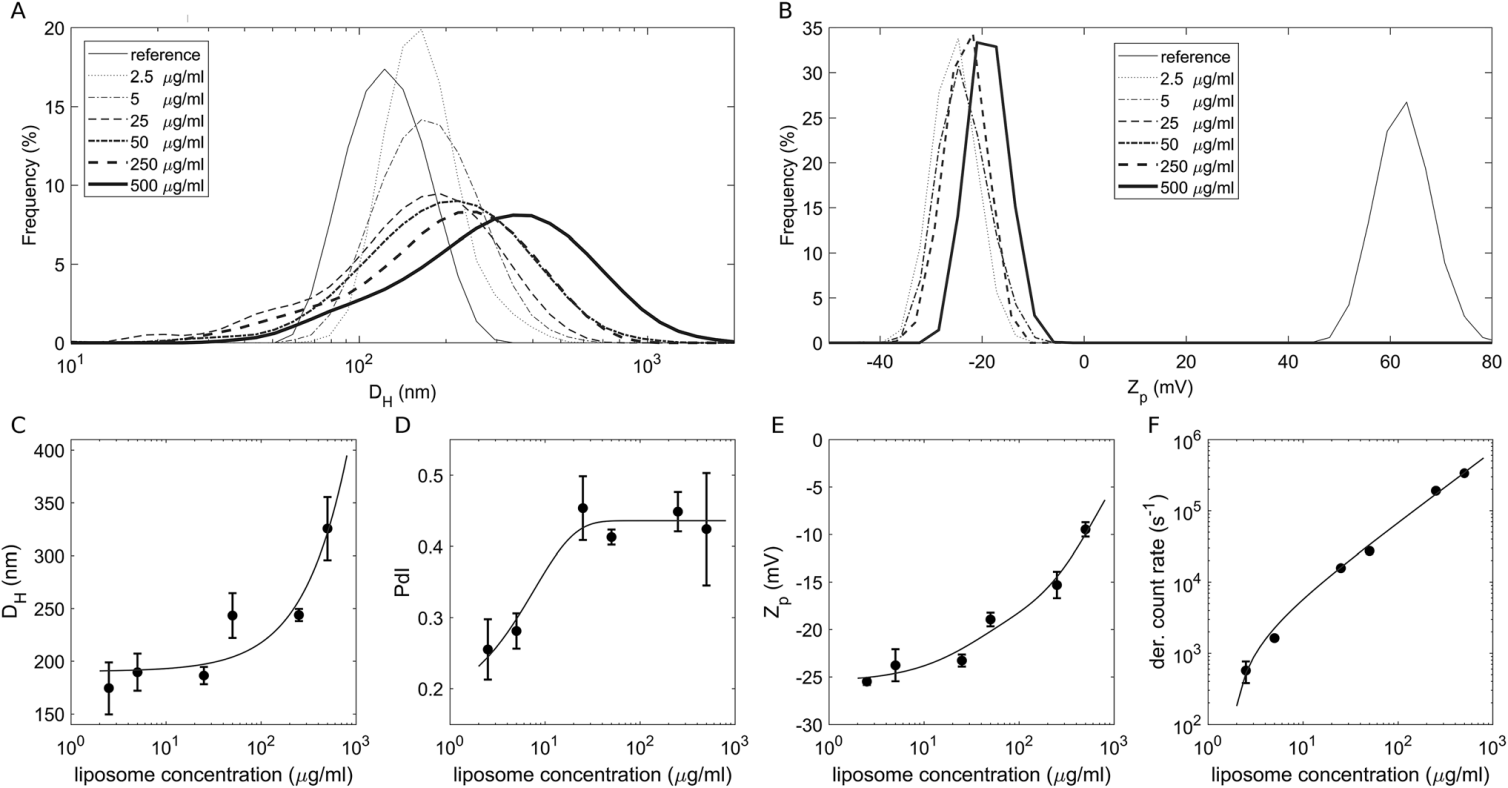
(A) Size distributions by intensity and (B) zeta potential (*Z*_p_) distributions of bare liposomes (reference curves) and liposome–BC complexes at a fixed liposome-to-plasma ratio and different sample concentrations. (C) Measured hydrodynamic diameter, (D) polydispersity index, (E) zeta potential and (F) photon count rate as functions of sample concentrations.

The corresponding zeta potential was cationic, with a distribution centred at 58.1 ± 3.9 mV. Upon incubation with human plasma, the samples exhibited a remarkable increase in size and the inversion of zeta potential from positive to negative values. These aspects represent two of the most common consequences of the formation of the BC on NPs. Indeed, the system's size is affected by the presence of the adsorbed biomolecular layer and the adsorbed plasma proteins (mainly anionic molecules at physiological pH) invert the original cationic surface charge of liposomes. In detail, size distributions of liposome–BC complexes were shifted and broader with respect to the reference curve of bare liposomes (Fig. 1A). Furthermore, as shown in Fig. 1C and D, the peak's location and polydispersity index increased with the sample concentration.

At low concentrations, the complexes were a few nanometres larger than bare liposomes. This finding is in agreement with numerous previous studies showing that the thickness of the BC can vary between a few to some tens of nanometers.^39^ However, beyond 50 μg mL^−1^, the measured hydrodynamic diameters were at least 100 nm larger than the reference one, thus suggesting that particle clustering has occurred. This aspect is particularly clear at 500 μg mL^−1^, at which the measured size is more than double that of bare liposomes (about 320 nm and 130 nm, respectively). As reported in spherical packing models, this remarkable increase in size is most likely due to particle aggregation in clusters made of two or more equal units. Thus, corona-coated liposomes were found to be small monomers at concentrations below 50 μg mL^−1^ and to form large complexes at concentrations higher than 250 μg mL^−1^. Besides the size and polydispersity of liposome–BC complexes, we also detected a non-linear trend of the zeta potential as a function of sample concentration. As Fig. 1E clearly shows, zeta potential values of liposome–BC complexes were negative and varied within a range of about [−25 mV, −10 mV]. In other words, the effects of concentration increase the surface charge of corona-coated liposomes, in a monotonic way. Finally, Fig. 1F presents the photon count per s that was proportional to the sample concentration, *i.e.* to the number of scattering objects in solution.

We studied the corresponding protein composition of the corona by 1D SDS-PAGE. Liposome–BC solutions were centrifuged at 18 620 rcf for 15 minutes at 4 °C (with a Z 216 MK centrifuge, Hermle, Germany) and then washed three times with phosphate buffered saline (PBS). The obtained pellets were suspended in 25 μL of Laemmli loading buffer 1×, boiled for 10 min at 100 °C and centrifuged at 18 620 rcf for 15 minutes at 4 °C. Then, supernatants were collected and diluted (1 : 20) before loading them on a stain free gradient polyacrylamide gel (4–20% TGX precast gels, Bio-Rad) and run at 100 V for about 150 min.

Finally, gel images were acquired with a ChemiDoc™ gel imaging system (Bio-Rad, CA, USA) and processed by means of custom MatLab scripts (MathWorks, MA, USA).

A representative gel image is shown in Fig. 2A; liposome–BC complexes were loaded from left to right at increasing sample concentrations. As expected, the total lane intensity depended on the absolute amount of the loaded sample. In this regard, a quantitative analysis is shown in Fig. 2B, where the total lane intensity is reported as a function of the concentration and exhibited an increasing sigmoid trend. However, the most interesting aspect of this experiment resides in the one-dimensional patterns associated with each sample. In fact, the intensity profile along a lane measures the molecular weight distribution of the corresponding corona. Fig. 2C depicts the normalized protein profiles that are roughly superimposable independent of the loaded absolute amounts. For each curve, a main narrow band was located at about 50 kDa, preceded by a distinct peak at 45 kDa and followed by a series of bands within the range [60 kDa, 90 kDa]. Secondary peaks were detected at about 20 kDa and 120 kDa. This behaviour was common to all the investigated samples, whose main differences are due to signal fluctuations at low concentrations, where a particularly small signal-to-noise ratio affected the resulting curves. These outcomes suggest that the protein composition of BCs was stable over a wide range of sample concentrations, which varied from 2.5 μg mL^−1^ to 500 μg mL^−1^.

**Fig. 2:**
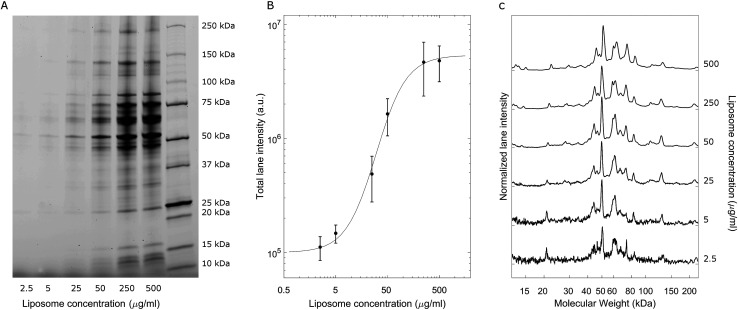
(A) Representative 1D SDS-PAGE image of liposome–BC complexes at a fixed liposome-to-plasma ratio (1 : 1 vol/vol) and different lipid concentrations. (B) Total lane intensity as a function of the sample concentration and (C) normalized curves depicting the corresponding electrophoretic patterns.

## Conclusions

**Fig. 3:**
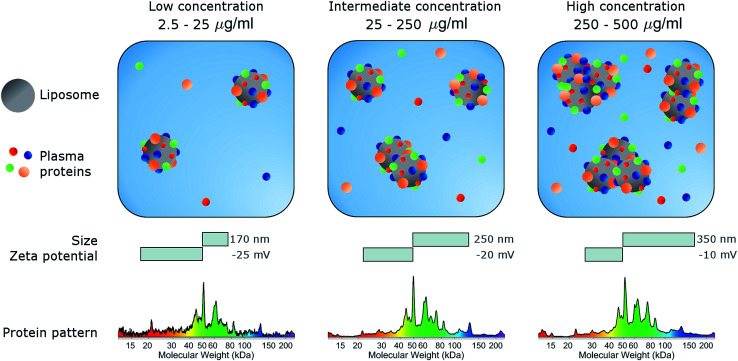
Schematic describing the effect of sample concentration on the biological identity of liposomes. Following exposure to plasma proteins, liposomes are coated with a biomolecular corona (BC). Liposome–BC complexes are larger in size than bare liposomes and negatively charged due to protein binding. The size and zeta-potential of liposome–BC complexes are largely dependent on the protein concentration. At a low protein concentration (0.05 mg mL^−1^) liposome–BC complexes are monomers and exhibit the most negative values of zeta-potential. With increasing concentration, aggregation occurs, and the zeta-potential becomes less negative.

In conclusion, here we studied the effects of concentration on the biological identity of a model liposomal system, in terms of size, aggregation state, zeta potential and corona composition. We found that the formation of a BC after exposure to human plasma altered the original physical–chemical properties of the chosen formulation, in a way that depended on the sample concentration (Fig. 3). Particularly, at low concentrations (<50 μg mL^−1^), corona-coated liposomes were slightly larger than the bare ones but for concentrations higher than 250 μg mL^−1^ large particle agglomeration occurred. Clear trends of the polydispersity index and zeta potential were also detected, whereas the protein composition of BC was stable over the explored range of concentrations. We therefore hope that this study will stimulate researchers to standardize characterization experiments. Current experimental procedures provide researchers with a macroscopically averaged composition of BC without addressing protein architecture at the particle surface.^40^ Future investigations will be aimed at exploring the effect of particle concentration on the distribution of exposed protein epitopes present across the corona surface.

## Conflicts of interest

There are no conflicts to declare.

## Supplementary Material
